# Clonal Hematopoiesis of Intermediate Potential in Atrial Fibrillation: A Critical View of Current Knowledge as a Springboard for Future Research

**DOI:** 10.3390/diagnostics15151915

**Published:** 2025-07-30

**Authors:** Elena Chatzikalil, Dimitris Asvestas, Stylianos Tzeis, Elena E. Solomou

**Affiliations:** 1 First Department of Pediatrics, National and Kapodistrian University of Athens Medical School, 11527 Athens, Greece; elenachatz@med.uoa.gr; 2 “Aghia Sofia” Children’s Hospital ERN-PeadCan Center, 11527 Athens, Greece; 3 Department of Cardiology, Mitera Hospital, 6, Erythrou Stavrou Str, Marousi, 15123 Athens, Greece; dimasvestas@gmail.com (D.A.); stzeis@otenet.gr (S.T.); 4 Department of Internal Medicine, University of Patras Medical School, 26500 Rion, Greece

**Keywords:** CHIP, atrial fibrillation, cardiovascular remodeling, *DNMT3A* mutation, *TET2* mutation

## Abstract

Clonal hematopoiesis of intermediate potential (CHIP) is the presence of a clonally expanded hematopoietic stem cell because of a mutation in individuals without evidence of hematologic malignancy, dysplasia, or cytopenia. Interestingly, CHIP is associated with a two-fold increase in cardiovascular risk, independently of traditional risk factors. Recent studies using deep-targeted sequencing have revealed that CHIP mutations, primarily *TET2* and *DNMT3A*, present a higher incidence in patients with AF compared to healthy controls. Moreover, the presence of the aforementioned mutations is positively correlated with the progression and the severity of the AF clinical course. Regarding the predisposition of AF, it has been proven that *TET2* and *ASXL1* mutations, and not *DNMT3A* mutation, are associated with higher interleukin-6 (IL-6) levels. IL-6 levels, being indices of cardiac remodeling, predispose to an elevated risk for AF in healthy subjects. Currently conducted research has focused on elaborating the mechanisms driving the association between AF and CHIP and on the evaluation of potential interventions to reduce the risk of AF development. The aims of our review are (i) to summarize published evidence regarding the presence of CHIP mutations as a contributor to AF severity and predisposition, and (ii) to highlight the potential benefits of investigating the correlations between CHIP and AF for AF-diagnosed patients.

## 1. Introduction

Cardiac arrhythmias represent a substantial health burden and are associated with increased risk of related complications in all age groups, including life-threatening strokes, heart failure, cardiac arrest, and sudden infant death [1,2,3]. Recent evidence suggests that cardiac arrhythmias affect approximately 1–5% of middle-aged populations, with their prevalence increasing by increasing age, especially in chronically ill populations [4,5,6,7,8]. Atrial fibrillation (AF) is the most common supraventricular cardiac arrhythmia, with an incidence of 1% in the general population and up to 10% in the elderly. AF is associated with increased mortality, accounting for up to 50% of cardiovascular deaths [2,9,10], and is considered a multifactorial disease, as its pathogenesis is influenced by several factors, including age, ethnicity, physical activity, and comorbidities (e.g., diabetes, hypertension) [11,12,13]. Chronic systemic and myocardial inflammation enhances structural remodeling and fibrosis of the myocardial tissue, leading to AF pathogenesis, while acute cardiac inflammation after being exposed to a triggering factor (e.g., post-vaccination) also leads to sometimes lethal cardiac arrhythmias, including AF [14,15,16]. AF pathogenesis involves an interaction between several initiating factors, manifesting as multiple rapidly firing ectopic foci within at least one pulmonary vein, along with abnormal atrial myocardial tissue capable of sustaining the arrhythmia [14]. Structural heart disease is the triggering factor behind many AF cases. It was shown that defects in molecular pathways are also involved in AF pathogenesis, leading to electrical conduction dysregulation, while human aging, as a state of chronic inflammation, is a potential contributor to the higher prevalence of AF in the elderly [17,18,19]. Epidemiological studies provide evidence that genetic factors play a crucial role in approximately 30% of patients with AF. Moreover, several studies propose a range of rare gene involvement (including *GJA1*, *GJA5*, *KCNQ1*, *LMNA*, *NUP155*, *JPH2*, *SYNE2*, and *GREM2*) associated with ionic channels, calcium-handling protein, fibrosis, conduction, and inflammation, in AF pathogenesis [20,21].

Clonal hematopoiesis of indeterminate potential (CHIP) is defined by the presence of somatic mutations with a variant allele frequency (VAF) of at least 2% in peripheral blood, which leads to the expansion of mutated clones in the absence of cytopenia, dysplastic hematopoiesis, and diagnostic criteria of hematologic malignancy [22,23]. CHIP is a predisposing state of hematological malignancies (13-fold increased risk), including monoclonal gammopathy of unknown significance (MGUS) and myelodysplastic syndrome (MDS), combined or not with other mutations or epigenetic alterations [24,25,26]. The majority of CHIP carriers remain asymptomatic; however, they have a 1.4-fold increased risk of mortality, although the annual risk of malignant development remains low [27]. Prior chemotherapy and radiotherapy are associated with CHIP development, mainly affecting DNA repair genes (e.g., *TP53*), leading to therapy-related myelodysplasia or leukemia, while bone marrow transplantation-derived CHIP mutations are associated with poor prognosis and higher mortality rates [28]. The expansion of mutant hematopoietic stem cells that lead to CHIP development is age-related, conferring risk for multiple diseases of aging, including not only hematologic cancers, but also cardiac diseases (myocardial infarction, arrhythmias, heart failure) [29,30,31,32]. The development of cardiac disease involves inflammatory cells derived from the cells of erythroid, myeloid, lymphoid, and megakaryocytic lineage, all of which originate from hematopoietic stem cells [33]. CHIP is an independent risk factor for cardiac disease and all-cause mortality [34,35]. Its involvement in cardiovascular disease is mainly suggested for atherosclerosis and heart failure, while individuals with VAF > 10% are at a higher risk of myocardial infarction, calcific aortic disease’s mortality and progression, and, generally, heart-failure related hospitalization [36,37,38,39].

The potential association between somatic mutations and AF pathogenesis has been largely studied within the last two decades, revealing pathogenic mutations in ion channel genes (*KCNQ1*) [40] and genes encoding junction proteins (*GJA5* and *GJA1*) [41,42]. Emerging evidence suggests that CHIP somatic mutations are a potential contributor to AF development [43,44]. Population-based studies and gene-specific analyses indicate that several CHIP mutations are associated with a higher incidence of AF, while their presence predisposes to AF-related adverse events [40]. However, several gaps regarding the specific role of CHIP in AF remain inadequately studied, as most real-world research has primarily focused on atherosclerosis and heart failure, with limited emphasis on AF. To date, only two reviews [43,44] have specifically addressed the aforementioned relationship, highlighting the lack of in-depth analysis of the shared risk factors at a clinical level, the limited number of in vivo prior studies, and the unexplored role of less common gene-specific subtypes. Given both the complexity in the management of AF [45] and the increasing availability of targeted next-generation sequencing with prediction utility in a variety of pre-neoplastic conditions [46,47], CHIP-direction and intervention strategies should be considered in AF patients. Defining the pathophysiologic correlations between CHIP and AF pathogenesis could significantly reduce the risk of severe complications in patients with AF. Herein, we provide a current state of the field regarding the identification and clinical impact of AF-related CHIP mutations, discussing the potential inclusion of these alterations in AF diagnostic and therapeutic approaches.

## 2. Pathophysiologic Interconnections Between CHIP and AF

### 2.1. The Role of Aging

Aging is considered the primary predictor of cardiovascular diseases, surpassing several traditional risk factors (including hypertension, dyslipidemia, and smoking) [48]. Aging leads to a gradual decline in tissue and organ function, along with the accumulation of somatic mutations [48]. A great variety of these somatic mutations, originating mostly in the bone marrow (and less in the heart and blood vessels), affect the cardiovascular system [49]. However, only a small subset of these mutations provides a selective advantage, leading to a clonal expansion and contributing to age-related conditions, including AF [49]. CHIP exhibits a similar age-dependent pattern, affecting 9.5% of people aged 70–79 years, 11.7 of those aged 80–89 years, and 18.4% of those over 90 years, while being rare in individuals under 40 years of age, demonstrating an annual increase of 6.7% and being associated with epigenetic aging [44]. This evidence combined suggests that CHIP is a potential mediator between aging-related biological changes and cardiovascular disease development [50]. As CHIP prevalence rises with age, specific driver mutations show distinct clonal patterns; mutations in *DNMT3* and *JAK2* tend to occur early, even during fetal development or childhood in some cases, while *SF3B1* and *SRSF2* mutations typically emerge later in adulthood [51]. Recently published research suggested that CHIP mutations are found more frequently in individuals with AF than in healthy controls, and they tend to increase patients’ vulnerability to AF complications [52]. However, the direct causal relationship between AF and CHIP remains uncertain, with age-related inflammation and telomere shortening in leucocytes being considered as key mediators, as discussed in the next sections of our review [44].

### 2.2. Inflammation: Macrophage Activity and Cytokine Storm Predisposing to Cardiovascular Risk and Thrombotic Events

**Figure 1:**
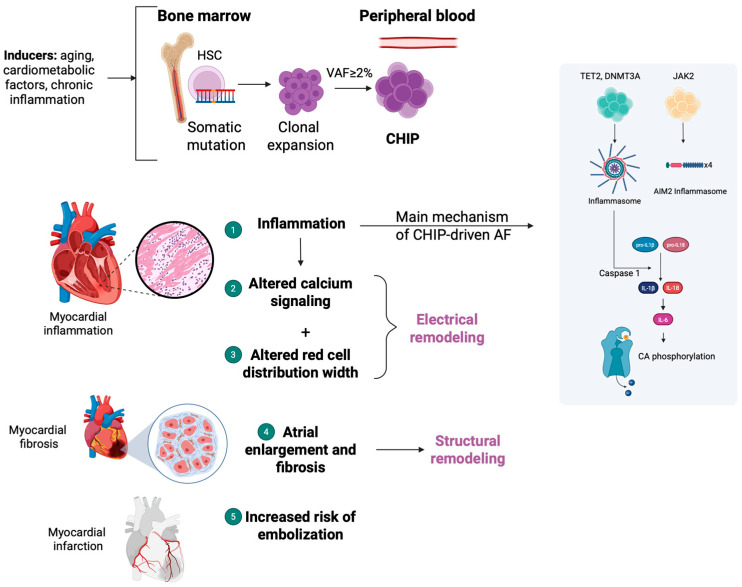
Pathophysiologic interconnections between CHIP and AF. The accumulation of age-related somatic mutations leads to the formation of clonal populations that lead to CHIP in the peripheral blood. Mutated hematopoietic cells infiltrate the bloodstream and myocardium, promoting atherosclerosis and adversely affecting cardiac function via several pathogenetic mechanisms, including atrial fibrosis, inflammation, and elevated red cell distribution width, resulting in structural and electrical remodeling of the heart. A pivotal mechanism interconnecting CHIP and AF is an inflammasome-mediated response, particularly through the interleukin-1/interleukin-6 signaling axis, which also causes abnormal calcium release, further enhancing electrical remodeling. The above mechanisms combined create a vicious cycle that promotes clonal expansion and the progression of AF. [AF: atrial fibrillation; AIM2: inflammation related gene absent in melanoma 2; CA: calcium; CA phosphorylation: CA-mediated phosphorylation; CHIP: clonal hematopoiesis of indeterminate potential; HSC: hematopoietic stem cells; IL-1: interleukin-1; IL-6: interleukin-6; IL-8: interleukin-8; VAF: variant allele frequency] (Figure created by Biorender software version 4.0, Toronto, Canada, publication license: # *KV28DAWCU5*).

The presence of CHIP mutations has been associated with an inflammatory pro-thrombotic state, which includes dysregulation of monocytes and macrophages, resulting in increased risk of cardiovascular complications in AF patients, and high risk of complications and mortality [53,54]. Chronic inflammation, which subsequently leads to endothelial dysfunction and thrombin generation, represents a potential association between AF-related cardiovascular risk and CHIP [55,56,57]. This statement initially stemmed from evidence associating significantly elevated cytokine levels with CHIP mutations, as a part of the investigation on how the inflammatory microenvironment leads to the development of neoplastic or pre-neoplastic conditions [58,59,60]. Cytokine levels are associated with monocyte activity regulation, macrophage formation, and atherosclerosis progression; thus, alterations in cytokine profiles and dysregulated monocytic and macrophage functions may cause vascular deformities [43,55,56,57]. In older individuals, elevated cytokines are significantly linked both to CHIP and AF, and notably, the NLRP3 inflammasome pathway plays a crucial role, activating caspase-1 in macrophages and leading to the conversion of pro-IL-1β and pro-IL-18 into active cytokines, stimulating IL-6 production [61,62] (Figure 1). These cytokines enhance changes in atrial electrophysiology via prolongation of action potentials and promoting calcium dysregulation (which is further dysregulated by inflammasome activation), with promoted arrhythmogenic activity being the final result of these processes [63,64] (Figure 1).

Investigating the role of inflammation as a link between CHIP and AF pathogenesis, the study of Fuster and his colleagues demonstrated a direct causal association between CHIP and atherosclerosis through a persistently inflammatory state, consisting of increased IL-1β levels and atherosclerotic pathogenesis in *TET2*-mutant mouse models [65]. Inhibition of IL-1β activity resulted in a significant reduction of the atherosclerotic potential, a finding that needs to be further evaluated by in vivo studies [65]. Another study in animal models reported that pharmacological inhibition of NLRP3 inflammasome in *TET2*-deficient mice resulted in decreasing the inflammatory state, lowering IL-1β, and ameliorating atherosclerosis and heart failure [66]. In the next years, research focused on both the two most common CHIP mutations (*TET2* and *DNMT3A*), demonstrating in vitro and in animal models that *TET2* deficiency upregulates the secretion of IL-1β, IL-6, and TNF-α [67,68], while *DNMT3A* deficiency was strongly associated with elevated cytokine (CXCL1, CXCL2, IL-6, and CCL5) levels in macrophages [69]. Importantly, individuals with germline *DNMT3* alterations have shown reduced monocyte IL-10 secretion. *DNMT3* activation has recently been considered a stimulator of antiviral responses in macrophages via histone deacetylase 9 [68,70]. Regarding *JAK2* mutations, it is suggested that they may influence macrophage functionality as well, inducing DNA replication and activating the inflammasome, subsequently worsening atherosclerosis [71]. These results combined suggest that a clonal hematopoiesis-driven pro-inflammatory state is a potential contributor to AF development.

### 2.3. Atrial Remodeling: Fibrotic Changes and Altered Calcium Handling

Left atrial enlargement is considered a risk factor and a consequence of AF, at the same time, creating a cycle in which AF promotes further atrial dilation [72,73]. Cardiac fibrotic changes, which increase with increasing age and are associated with extracellular matrix buildup, are also main contributors to atrial remodeling [74,75]. The mechanisms behind cardiac fibrosis are complex and not yet fully elucidated; however, inflammation and shortened leukocyte telomere length are considered as main pathophysiological mechanisms, while disturbances in calcium signaling may contribute to electrical atrial remodeling [76,77]. Chronic inflammation, as indicated by elevated CRP and IL-6 levels, leads to atrial remodeling and fibrosis mediated by macrophage-derived osteopontin (SPP1) [78,79,80]. Shortened leukocyte telomere length has been associated with higher CHIP prevalence, as longer telomeres may promote cell division and increase somatic mutation risk [81]. Conversely, CHIP mutations, especially in genes like *TET2* and *ASXL1*, can accelerate leukocyte telomere length shortening, leading to greater atrial dilation and progression of atrial fibrosis [19,76]. Several signaling pathways may be involved in these processes, mainly EGFR/Akt signaling, which disrupts electrical impulse propagation and fosters re-entrant circuits, finally worsening AF [74].

Dysregulation of calcium signaling is a crucial link between CHIP and AF. In individuals carrying *TET2* mutations, impaired calcium handling in heart cells, which occurs due to the activation of NLRP3 inflammasome, has been demonstrated [82,83]. In *ΤΕΤ2*-deficient mouse models, increased calcium flux in the sarcoplasmic reticulum was observed in one study, leading to abnormal calcium release into the cardiac cells’ cytosol, and further promoting AF [83]. Moreover, cardiomyocytes from *TET2*-deficient mice have demonstrated prolonged calcium transient and release, contributing to electrical remodeling and increased AF risk [83]. Several other studies support the role of cardiac macrophages in electrical cardiac functionality and arrhythmia development [84,85,86,87], while calcium dysregulation is considered to be worsened by inflammatory molecules (IL-1β and IL-6) in *TET2*-deficient macrophages [39]. Several in vivo studies confirm that cultures of atrial cardiomyocytes with *TET2*-deficient macrophages cause reduced serum calcium and impaired calcium transients, along with findings of studies on mouse models [39,83].

### 2.4. Other Secondary Potential Mechanisms (Thrombophilia, Elevated Red Cell Distribution Width)

Thromboembolism is a common complication of AF, as previously referred to herein [88]. CHIP is strongly associated with pro-thrombotic states, potentially elevating the risk of thromboembolic events in AF patients [89]. CHIP mutations, mainly *JAK2*, are linked with elevated thrombotic risk, increasing megakaryocyte activity, enhancing platelet reactivity through hypersensitive thrombopoietin (MPL) receptors, and raising levels of procoagulant microvesicles; all these represent mechanisms that enhance coagulation and blood clotting [90,91]. In addition to *JAK2*, mutations in *TET2* and *DNMT3A* also contribute to CHIP-related hypercoagulability [83]. *TET2* mutations lead to increased inflammatory cytokines like IL-1β and IL-6, worsening endothelial dysfunction and thrombin production [92]. This pro-inflammatory environment interacts with coagulation pathways, creating conditions conducive to thromboembolic events [93,94].

Red cell distribution width (RDW) reflects the variability in erythrocyte size and is closely related to hematopoietic dysfunction [95]. Elevated RDW is a strong predictor of increased mortality and poor prognosis in AF patients [96]. In non-valvular AF, RDW levels below 13.9% strongly indicate thromboembolic events, with predictive accuracy increasing at older ages [97]. Additionally, a meta-analysis reported a significant connection between elevated RDW and AF pathogenesis, while, at the same time, RDW is the only hematological parameter that is found consistently elevated in CHIP carriers, potentially indicating underlying disruptions in erythropoiesis or hematopoiesis in general, which both represent characteristic features of CHIP [98,99].

## 3. Initial Observations Supporting the Association Between CHIP and AF and Shared Risk Factors

Recently reported clinical evidence highlights the significant interplay between CHIP alterations and AF pathogenesis, complications, and risk stratification. A population-based study by UK Biobank on over 200,000 individuals found a modest 1.09-fold increased risk of AF in those with CHIP [100]. Several mutations, mainly in TET2 and ASXL1, are associated with increased AF risk, at percentages 1.18- and 1.33-fold, respectively [53,100,101]. Moreover, CHIP mutations are associated with specific disease subtypes in AF patients, namely postoperative AF (POAF) and in-hospital AF, predisposing individuals of all ages carrying CHIP mutations for undergoing aortic valve replacement to have a 3.5-fold higher risk of POAF [62,101,102]. The prevalence of AF is also significantly higher among CHIP carriers who underwent cardiac surgery and stem cell transplantation, especially those with larger clones (variant allele frequency above 10%). These individuals also tend to experience higher recurrence rates. While a dose–response relationship between CHIP clone size and AF risk has been hypothesized, causality remains uncertain due to potential confounding factors and shared aging-related pathways [103,104]. Moreover, several risk factors that predispose both to CHIP and AF have been identified, including age, lifestyle, and cardiometabolic comorbidities [44].

Increasing aging is the main predisposing factor of cardiovascular disease, surpassing traditional risk factors (e.g., hypertension, smoking), and leading to dysregulation of several mechanisms of myocardial functionality and to the acquisition of somatic mutations, as well [105,106]. A large number of mutations that affect the cardiovascular system originate from the bone marrow, though only a minority of them confer a growth advantage that leads to clonal expansion and accelerates age-related conditions, including AF [96]. A significant increase in AF with age is observed in several population studies, specifically from approximately 2% in the general population to 10–12% in individuals over 80 years of age [107,108]. An association between CHIP and epigenetic aging has been demonstrated, with a potential link with cardiac disease development [43]. Specifically, *TET2* mutations that can occur at any year of age but are most common in individuals above 80 years of age, are likely to influence both CHIP and AF pathogenesis, with CHIP mutations becoming more prevalent with age and found more frequently in patients with AF compared to healthy individuals [109,110]. The mechanisms behind these correlations have not been fully identified; however, factors like age-related inflammation and telomere shortening are suggested to play a crucial role [43]. Moreover, lifestyle factors, including smoking, cardiometabolic syndrome (including related comorbidities, e.g., diabetes, PCOS) [111], and a high-fat diet, not only predispose to cardiac disease, but also have a 16% increased risk of CHIP, particularly with *ASXL1* mutations, and this risk persists even after quitting [112,113]. Diet similarly influences these conditions. Low vegetable or high meat consumption is linked to a 25% higher risk of CHIP and increased AF susceptibility, indicating that dietary choices may impact both through common mechanisms [112,114].

In more detail regarding cardiometabolic comorbidities, patients with AF and CHIP mutations present a higher prevalence of hypertension, while *TET2* deficiency, due to *TET2* somatic mutations identified in human tissues via deep-targeted sequencing, has been demonstrated in population-based studies to be an aggravating factor for cardiac dysfunction, NLRP3 inflammasome activation, and sodium retention, effects that can be considered potential therapeutic targets in AF patients [58,115]. Obesity and diabetes are also main contributors to both conditions, while higher body mass index (BMI) and waist-to-hip ratios are associated with increased CHIP prevalence [100,112]. In diabetic patients, CHIP independently increases cardiovascular disease risk by 21% [65]. Conversely, AF patients with CHIP (especially with *TET2* and *ASXL1* alterations) face a 23% higher diabetes risk [116]. Studies on animal models have reported potential links between obesity, particularly leptin deficiency, and increased CHIP levels and inflammation, underscoring the interplay between metabolic health and CHIP in cardiovascular disease [117].

## 4. Clinical Studies and Real-World Data

Collectively, several recent population-based studies have demonstrated the potential link between CHIP and AF, highlighting the potential of targeting CHIP mutations as a part of AF therapy and prevention. A study on over a thousand AF individuals reported CHIP mutations in 23.6% of AF patients and 10.7% in controls, using deep-targeted sequencing of 24 CHIP-associated genes with a VAF of ≥2% as a threshold [49]. Specifically, for TET2 mutations, this study showed that they were significantly more frequent in AF patients with an adjusted odds ratio of 1.65. Individuals with AF and CHIP demonstrated worse clinical phenotype, including longer AF duration, increased left atrial size, and higher diastolic dysfunction [49]. Moreover, in this cohort, AF patients with CHIP presented a 1.32-fold risk of severe adverse events (heart failure and stroke), while the presence of CHIP mutations was an independent risk factor of mortality [58]. Another population-based study on *TET2* mutations, using evidence from both in vivo studies of a patient cohort (358,000 participants in total) and murine models, showed that CHIP (VAF ≥ 2%) predisposed to AF development with an 11% increased risk, with *TET2* mutations conferring the highest risk [83]. Interestingly, *TET2*-deficient mouse models demonstrated that inflammation via the NLRP3 inflammasome and calcium handling abnormalities in atrial cells contributed to AF susceptibility, findings that were further proved by the decreased arrhythmia incidence after NLRP3 inhibition [74].

CHIP mutations in AF patients were further investigated using the large patient Biobank’s data of over a million AF individuals. To begin with, a large UK Biobank study on over 410,000 middle-aged adults without baseline arrhythmias, reported, by using whole-exome sequencing and identifying CHIP with VAF ≥ 2% (any CHIP) and ≥10% (large CHIP), that CHIP was an independent risk factor for the development of cardiac arrhythmias, especially the large clones [101]. *TET2* and *ASXL1* mutations are common CHIP mutations predisposing to cardiac arrest, with inflammation and myocardial remodeling proposed as the main pathogenetic mechanisms [101]. Another population-based study using the ARIC and UK Biobank studies reported similar results for *TET2* and *ASXL1* mutations, specifically suggesting that *TET2* mutations were associated with elevated IL-6, indicating systemic inflammation, while *ASXL1* mutations correlated with markers of cardiac remodeling, such as increased troponin T levels and left ventricular mass [19]. *ASXL1* mutations have also been studied in a high-risk cohort consisting of cardiac catheterized patients and have been shown to be significantly different (2-fold increased) [19]. Screening for the aforementioned CHIP mutations is not yet part of standard AF practice in patients’ cohorts, primarily due to the absence of proven interventions to mitigate the increased cardiovascular risk associated with these mutations. Further research, including animal studies and clinical trials, is essential to convert this expanding knowledge into targeted, personalized strategies for preventing and managing AF.

## 5. Driver Mutations Involving CHIP and AF: A Brief Sum-Up of Their Potential Distinct Role

The most commonly observed driver mutations are *DNMT3A*, *TET2*, and *ASXL1*, followed by several other mutations, including *JAK2*, *PPM1D*, *TP53*, *SF3B1*, and *SRSF2*. Current knowledge on the role of each gene mutation in AF pathogenesis is discussed in the next sections.

### 5.1. DNMT3A Mutation

DNMT3A is an epigenetic regulator that encodes a methyltransferase controlling gene silencing via CpG methylation, playing a crucial role in hematopoietic stem cell (HSC) self-renewal and differentiation [118,119]. *DNMT3A* mutations represent over 50% of identified CHIP mutations [110]. In the UK Biobank, over 3.4% of individuals carry these mutations [19,58]. Mechanistically, *DNMT3A* mutations, mainly detected in peripheral blood, activate monocytes and CD8+ T cells, triggering the NLRP3/IL-1/IL-6 inflammatory axis and promoting resistance to apoptosis via interferon-gamma (IFN-γ) [120,121,122]. They also increase CXCL chemokine expression and activate pathways like RASSF1A-ERK1/2 in hematopoietic stem and progenitor cells, which can contribute to myocardial fibrosis [121,122,123]. The effect of *DNMT3A* mutations on cardiovascular disease is not yet fully elucidated; however, recent evidence demonstrates its association with many diseases, including atherosclerosis, heart failure, or peripheral artery disease, while in atrial fibrillation, as previously reported herein, it presents a rather limited impact [40]. This lack of correlation is partially related to the absence of associations between *DNMT3A* mutations and mean leukocyte telomere length [40].

In more detail, clinical studies on stem cell transplant recipients or individuals with familial *DNMT3A* mutations have demonstrated several correlations with cardiac abnormalities, suggesting that they are explained mainly by secondary factors (transplant-related stress and inflammation) rather than the mutation itself; therefore, although *DNMT3A* mutations do not directly cause AF, they affect its pathogenesis through inflammatory pathways and signaling pathways [124,125]. A recent study showed that *DNMT3A* possibly contributes to recurrent AF in elderly populations via the activation of the PI3K-Akt signaling pathway, in combination with the downregulation of miR-200b [126]. These observations, however, present limitations, considering that the abnormal levels of *DNMT3A*, miRNAs, and PI3K-Akt molecules in circulating serum may be influenced by various factors, making it difficult to establish a direct link to AF [40]. Further in vitro and in vivo evidence is needed to confirm these observations.

### 5.2. TET2 Mutation

*TET2*, the second most common CHIP mutation, encodes a dioxygenase involved in DNA methylation, playing an important role in epigenetic regulation and hematopoietic processes [127,128]. In more detail, *TET2* mutations are detected in the peripheral blood of 5–10% of adult individuals older than 65 years of age and are associated with myeloid expansion and innate immunity dysregulation, further contributing to several diseases, including leukemia and cardiovascular disease [129,130]. In mouse models, it has been shown that *TET2* deficiency enhances myeloid differentiation via enhancing hematopoietic stem cell self-renewal, with bone marrow dysfunction and extramedullary hematopoiesis presenting as further consequences [131]. Epidemiological studies address a link between *TET2* mutations and an increased risk of AF, demonstrating associations between accelerated atherosclerosis, myocardial fibrosis, cardiac arrest, and adverse cardiac remodeling, with *TET2* alterations [44]. *TET2* gene alterations promote AF through structural and electrical atrial remodeling, with the main mechanisms being the decreased refractory periods and the enlarged left atrium, as observed in studies in animal models [83]. The activation of inflammatory pathways by *TET2* mutations, specifically the NLRP3/IL-1/IL-6 axis, and the subsequent dysregulated cytokine production in macrophages disrupts calcium signaling and cellular homeostasis, leading to further cardiac dysfunction and predisposing to AF development [83]. Worth noting, a recent study in *TET2*-mutant patients treated with IL-1β inhibitor canakinumab reports decreased cardiovascular events in these patients, highlighting the potential involvement of *TET2* mutations in cardiac disease via inflammatory pathways, as well as the therapeutic role of targeting inflammation [44,132].

### 5.3. ASLX1 Mutation

*ASLX1* is the third most common CHIP mutation, mainly presenting as a frameshift or nonsense variant in the terminal gene exon. It has a crucial role in epigenetic regulation by promoting gene activation through chromatin interactions [133]. *ASLX1* mutations are mainly encountered in older individuals with chronic inflammation (e.g., prior HIV infection), where inflammation is considered a main driver of clonal expansion, highly correlated with mortality rates [134,135]. The involvement of *ASLX1* mutations in AF has not yet been fully described, with current evidence addressing a 22% elevated risk per 10% increase in the size of clones; however, other studies report no significant correlations, possibly due to the different VAF thresholds [19,83]. At a pathophysiologic level, *ASLX1* mutations may be involved in cardiac remodeling via inflammatory pathways, as has been suggested by studies in mouse models correlating *ASLX1* with elevated macrophage infiltration and pro-inflammatory cytokine levels [122]. At the clinical level, this generally inflammatory environment is associated with adverse cardiac phenotypes, including increased serum troponin, increased left ventricular mass index, and elevated N-terminal pro-BNP, factors indicating ongoing cardiovascular damage and heart-related pathologies [136].

### 5.4. Other Driver Mutations (JAK2, TP53, PPM1D, Spliceosome)

*JAK2* encodes a tyrosine kinase involved in receptor signaling for hematopoietic cytokines, including growth hormone and prolactin, and also plays a crucial role in *TET2* phosphorylation [137,138]. *JAK2* mutations can be detected using both peripheral blood and bone marrow samples. The *V617F* mutation, which represents the most common *JAK2* variant, is encountered mostly in individuals of a younger age and is strongly associated with clonal hematopoiesis, with a detection rate of approximately 3% [139]. *JAK2* mutations significantly increase the risk of coronary heart disease, mainly via platelet vulnerability enhancement [139]. Although the association between *JAK2* mutations and AF has not been much investigated, pathophysiologic mechanisms previously analyzed herein create a potential association: *JAK2* mutations may lead to atrial remodeling and fibrosis via activating the inflammasome and NLRP3 pathway, being involved in AF pathogenesis [139,140]. Primary results from animal studies show that *JAK2* inhibition decreases atrial fibrosis, preventing electrical and mechanical remodeling; however, real-world data from further cohort studies are needed to confirm this hypothesis [44]. *TP53*, *PPM1D*, and spliceosome gene mutations (*SF3B1* and *SRSF2*) are less common CHIP driver mutations, which lead to genomic instability (*TP53*), regulate hematopoietic stem cell renewal (*TP53* and *PPM1D*), are essential for *RNA* splicing and gene regulation (*SF3B1* and *SRSF2*), or are associated with high mortality rates and poor chemosensitivity (*PPM1D*), all being strongly associated with hematologic malignancies [40]. Their role on AF is rather under-investigated; however, UK Biobank suggests that *TP53* and *PPM1D* may elevate arrhythmia, and a recent cohort study on individuals carrying *SF3B1* mutations showed a 1.73-fold increased risk of arrhythmia for *SF3B1* mutations [141]. Notably, *SF3B1* is considered a common genetic alteration among diabetics who exhibit a 2.5-fold increased cardiovascular risk, which highlights the need for further investigation on its roles [142].

A brief sum-up of research findings for the role of the aforementioned CHIP mutations in AF pathogenesis is presented in Table 1.

## 6. Discussion and Future Directions

**Figure 2:**
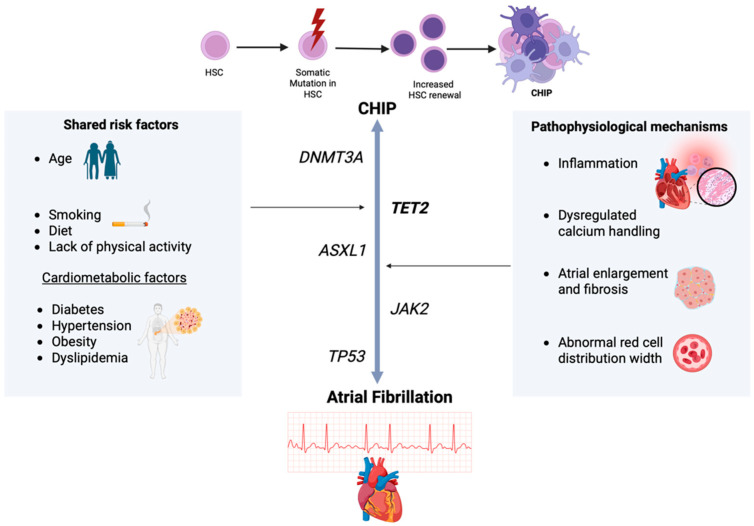
Illustration of shared risk factors (age, lifestyle factors, and cardiometabolic factors) and pathophysiological links between CHIP and AF, along with the identified CHIP mutations playing a role in AF pathogenesis. [CHIP: clonal hematopoiesis of intermediate potential, HSC: hematopoietic stem cell] (Figure created by Biorender software version 4.0., Toronto, Canada, publication license: # U28DA6J3E).

CHIP and AF are two distinct entities sharing common risk factors and pathophysiological mechanisms (Figure 2); however, a direct causal link between them has not yet been confirmed. Chronic inflammatory state is considered to be a central mechanism in oncogenesis and in cardiac disease, dysregulating calcium homeostasis and enhancing abnormal electrical activity, while other mechanisms, mainly atrial enlargement, serve as potential contributors, as well [40,143,144,145,146]. The exact role of the different driver CHIP mutations on AF pathogenesis varies. The strongest association to AF is served by *TET2* mutations, while *DNMT3A*, despite being the most common CHIP mutation, generally presents no significant association with AF, except in conditions of chronic inflammatory disease or post-transplantation stress [43,124,125,126]. The distinct roles of rarer CHIP mutations (*ASXL1*, *JAK2*, *TP53*, *PPM1D*, and spliceosome components) might also influence AF [19,44,74,122,136,139,140,141]. The emerging effect of inflammation, and especially inflammasome activity, in *CHIP-TET2*/*DNMT3* association, suggests the promising role of targeting the inflammasome in order to reduce inflammation and subsequently decrease AF complications and mortality risk [39,147,148]. Colchicine, canakinumab, and IL-6 blockers are anti-inflammatory drugs being tested in currently conducted clinical trials on individuals carrying CHIP mutations [149]. Hypomethylating agents, mainly *JAK2* inhibitors, antifibrotic medication, mainly sodium-glucose co-transporter-2 (SGLT2) inhibitors, and GLP-1 receptor agonists are also potential inhibitors of AF progression; however, further research is needed to confirm their efficacy in CHIP-related AF [139,150]. Regarding SGLT2 inhibitors, their protective effect against cardiovascular disease has been confirmed in recent studies, which indicate a possible pathophysiologic interconnection between AF and heart failure with reduced ejection fraction [150]. However, other studies show contradictory results, suggesting that SGLT2 inhibitors do not decrease the risk of AF occurrence, regardless of other factors (type of therapeutic evaluation, patients’ follow-up, and patients’ demographic and clinical characteristics). Further research is needed to establish an exact correlation [151,152,153].

Despite the significant progress in understanding the complex correlations between CHIP and AF, in vivo investigation faces several limitations, including the variability in sequencing methods (mainly the low sensitivity of whole exome sequencing in detecting low VAF clones and different VAF thresholds in different studies) and the lack of diversity of the studied cohorts, as most studies analyzed herein are predominantly conducted in European populations [19,39,40,74,112,114,115,116,125,128,129,130]. In the near term, clinical application of current knowledge, in terms of applying the aforementioned evidence on further exploring and targeting CHIP mutations (mainly *TET2* and *JAK2*) as diagnostic markers and therapeutic targets of the medication previously described (mainly hypomethylating agents), would be of a great interest for better establishing the role of CHIP mutations in AF patients. While anti-inflammatory therapies targeting NLRP3 mutations show potential in mitigating AF, further research is needed to evaluate their efficacy across various CHIP mutations and to determine the utility of CHIP as a predictive biomarker for classifying AF subtypes and related complications. Furthermore, exploring the dose–response relationship between the size of VAF clones and AF progression could further guide AF clinical management and prevention in individuals carrying CHIP mutations [39]. It would also be of great importance to incorporate translational and epigenomic research techniques—fundamental tools in ongoing investigations of CHIP mutations, leukemia, and other cancers—to uncover the indirect roles of CHIP in AF pathogenesis [154,155,156,157]. Chromatin immunoprecipitation followed by sequencing (ChIP-seq) is a novel technique that allows genome-wide analysis of histone modifications, enhancer activity, and chromatin states, providing evidence into how the epigenome influences cell identity, development, lineage specification, and disease, and could play a role in AF pathogenesis, especially for common CHIP mutations for which data regarding AF pathogenesis are scarce [158,159]. Other advanced genomic techniques, including polygenic risk scores, Mendelian randomization, and PheWAS, are considered promising for covering the aforementioned research gap in establishing optimal VAF thresholds for different populations in the near term. Another issue to be addressed is the identification of the mechanistic pathways linking specific CHIP mutations, particularly those involving the inflammasome, with inflammation and AF, which are currently not fully understood. Overall, integrating mechanistic insights with clinical, translational, and epigenetic research is the key to moving from correlation to causation, enabling personalized treatment strategies for AF in CHIP carriers.

## 7. Conclusions

In conclusion, emerging evidence strongly indicates CHIP mutations, especially *TET2*, are a promising risk factor for AF development. Recent findings analyzed herein underscore the potential advantage of CHIP mutation screening in AF patients, as well as the role of cardiovascular surveillance in individuals with CHIP mutations. Despite promising evidence from current studies, CHIP evaluation is not yet a part of standard cardiology practice, mainly due to the absence of established interventions mitigating the elevated CHIP-driven cardiovascular risk. Future research is essential for clarifying shared risk factors and signaling pathways between CHIP and AF, further aiming to develop CHIP-targeted therapeutic options, converting the expanding knowledge described in this review into targeted strategies for preventing and managing AF.

## Figures and Tables

**Table 1:** Driver CHIP mutations with a potential clinical impact on AF pathogenesis. [AF: atrial fibrillation; CHIP: clonal hematopoiesis of intermediate potential].

References	Driver CHIP Mutations	Pathophysiology	Clinical Impact on AF
[124,125,126,127]	*DNMT3*	CpG methylation, regulates HSC self-renewal and differentiation	No significant correlation. Worsening AF in special patient groups: the elderly, cases of chronic inflammation, and/or post-transplantation.
[129,130,132]	*TET2*	DNA methylation, regulates HSC self-renewal and differentiation at deficient state, promotes myeloid expansion	Strong correlation with AF development. Increased AF risk.
[19,83,122,136]	*ASXL1*	Regulates epigenetic processes via chromatin-binding proteins	Limited data for its association with AF pathogenesis.
[139,140]	*JAK2*	Tyrosine kinase activity	Mildly elevated risk for AF via the inflammasome pathway.
[44,45,46,47,48,49,50,51,52,53,54,55,56,57,58,59,60,61,62,63,64,65,66,67,68,69,70,71,72,73,74,75,76,77,78,79,80,81,82,83,84,85,86,87,88,89,90,91,92,93,94,95,96,97,98,99,100,101,102,103,104,105,106,107,108,109,110,111,112,113,114,115,116,117,118,119,120,121,122,123,124,125,126,127,128,129,130,131]	*TP53*	Regulates DNA damage repair	May elevate arrhythmias (including AF) in some cases.
[40,130]	*PPM1D*	Regulates DNA damage repair	May elevate arrhythmias in some cases.
[142]	*SF3B1*	Regulates mRNA splicing	A study reports increased AF incidence in patients with *SF3B1* mutations.

## Data Availability

All data are retrieved from Pubmed and Scopus.

## References

[1] Chugh S.S., Havmoeller R., Narayanan K., Singh D., Rienstra M., Benjamin E.J., Gillum R.F., Kim Y.-H., McAnulty J.H., Zheng Z.-J. (2014). Worldwide Epidemiology of Atrial Fibrillation: A Global Burden of Disease 2010 Study. Circulation.

[2] Khatib S.M., Stevenson W.G., Ackerman M.J., Bryant W.J., Callans D.J., Curtis A.B., Deal B.J., Dickfeld T., Field M.E., Fonarow G.C. (2018). AHA/ACC/HRS Guideline for Management of Patients with Ventricular Arrhythmias and the Prevention of Sudden Cardiac Death: Executive Summary: A Report of the American College of Cardiology/American Heart Association Task Force on Clinical Practice Guidelin. J. Am. Coll. Cardiol..

[3] Berdowski J., Berg R.A., Tijssen J.G.P., Koster R.W. (2010). Global Incidences of Out-of-Hospital Cardiac Arrest and Survival Rates: Systematic Review of 67 Prospective Studies. Resuscitation.

[4] Diederichsen S.Z., Xing L.Y., Frodi D.M., Kongebro E.K., Haugan K.J., Graff C., Højberg S., Krieger D., Brandes A., Køber L. (2023). Prevalence and Prognostic Significance of Bradyarrhythmias in Patients Screened for Atrial Fibrillation vs Usual Care: Post Hoc Analysis of the LOOP Randomized Clinical Trial. JAMA Cardiol..

[5] Sajadieh A., Nielsen O.W., Rasmussen V., Hein H.O., Frederiksen B.S., Davanlou M., Hansen J.F. (2006). Ventricular Arrhythmias and Risk of Death and Acute Myocardial Infarction in Apparently Healthy Subjects of Age≥ 55 Years. Am. J. Cardiol..

[6] Wolf P.A., Mitchell J.B., Baker C.S., Kannel W.B., D’Agostino R.B. (1998). Impact of Atrial Fibrillation on Mortality, Stroke, and Medical Costs. Arch. Intern. Med..

[7] Khurshid S., Choi S.H., Weng L.-C., Wang E.Y., Trinquart L., Benjamin E.J., Ellinor P.T., Lubitz S.A. (2018). Frequency of Cardiac Rhythm Abnormalities in a Half Million Adults. Circ. Arrhythm. Electrophysiol..

[8] Delaporta P., Chatzikalil E., Ladis V., Moraki M., Kattamis A. (2023). Evolving Changes in the Characteristics of Death in Transfusion Dependent Thalassemia in Greece. Blood.

[9] Myerburg R.J., Junttila M.J. (2012). Sudden Cardiac Death Caused by Coronary Heart Disease. Circulation.

[10] Goldberger J.J., Buxton A.E., Cain M., Costantini O., Exner D.V., Knight B.P., Lloyd-Jones D., Kadish A.H., Lee B., Moss A. (2011). Risk Stratification for Arrhythmic Sudden Cardiac Death: Identifying the Roadblocks. Circulation.

[11] Glezeva N., Baugh J.A. (2014). Role of Inflammation in the Pathogenesis of Heart Failure with Preserved Ejection Fraction and Its Potential as a Therapeutic Target. Heart Fail. Rev..

[12] Mesquita T., Lin Y., Ibrahim A. (2021). Chronic Low-grade Inflammation in Heart Failure with Preserved Ejection Fraction. Aging Cell.

[13] Scott L., Li N., Dobrev D. (2019). Role of Inflammatory Signaling in Atrial Fibrillation. Int. J. Cardiol..

[14] Leventopoulos G., Koros R., Travlos C., Perperis A., Chronopoulos P., Tsoni E., Koufou E.-E., Papageorgiou A., Apostolos A., Kaouris P. (2023). Mechanisms of Atrial Fibrillation: How Our Knowledge Affects Clinical Practice. Life.

[15] Lagousi T., Papadatou I., Strempas P., Chatzikalil E., Spoulou V. (2022). Paving the Way Towards Precision Vaccinology: The Paradigm of Myocarditis After Coronavirus Disease 2019 (COVID-19) Vaccines. Clin. Infect. Dis..

[16] Bhuiya T., Shah P.P., Lau W.H., Park T., Munshi R.F., Hai O., Zeltser R., Makaryus A.N. (2024). Emergence of Atrial Fibrillation and Flutter in COVID-19 Patients: A Retrospective Cohort Study. Healthcare.

[17] Papathanasiou K.A., Giotaki S.G., Vrachatis D.A., Siasos G., Lambadiari V., Iliodromitis K.E., Kossyvakis C., Kaoukis A., Raisakis K., Deftereos G. (2021). Molecular Insights in Atrial Fibrillation Pathogenesis and Therapeutics: A Narrative Review. Diagnostics.

[18] Huang J., Wu B., Qin P., Cheng Y., Zhang Z., Chen Y. (2023). Research on Atrial Fibrillation Mechanisms and Prediction of Therapeutic Prospects: Focus on the Autonomic Nervous System Upstream Pathways. Front. Cardiovasc. Med..

[19] Saadatagah S., Naderian M., Uddin M., Dikilitas O., Niroula A., Schuermans A., Selvin E., Hoogeveen R.C., Matsushita K., Nambi V. (2024). Atrial Fibrillation and Clonal Hematopoiesis in TET2 and ASXL1. JAMA Cardiol..

[20] Campuzano O., Perez-Serra A., Iglesias A., Brugada R. (2016). Genetic Basis of Atrial Fibrillation. Genes Dis..

[21] Feghaly J., Zakka P., London B., Macrae C.A., Refaat M.M. (2018). Genetics of Atrial Fibrillation. J. Am. Heart Assoc..

[22] Marnell C.S., Bick A., Natarajan P. (2021). Clonal Hematopoiesis of Indeterminate Potential (CHIP): Linking Somatic Mutations, Hematopoiesis, Chronic Inflammation and Cardiovascular Disease. J. Mol. Cell. Cardiol..

[23] Hoermann G., Greiner G., Griesmacher A., Valent P. (2020). Clonal Hematopoiesis of Indeterminate Potential: A Multidisciplinary Challenge in Personalized Hematology. J. Pers. Med..

[24] Mailankody S., Pfeiffer R.M., Kristinsson S.Y., Korde N., Bjorkholm M., Goldin L.R., Turesson I., Landgren O. (2011). Risk of Acute Myeloid Leukemia and Myelodysplastic Syndromes after Multiple Myeloma and Its Precursor Disease (MGUS). Blood.

[25] van de Donk N.W.C.J., Palumbo A., Johnsen H.E., Engelhardt M., Gay F., Gregersen H., Hajek R., Kleber M., Ludwig H., Morgan G. (2014). The Clinical Relevance and Management of Monoclonal Gammopathy of Undetermined Significance and Related Disorders: Recommendations from the European Myeloma Network. Haematologica.

[26] Chatzikalil E., Arvanitakis K., Filippatos F., Diamantopoulos P.T., Koufakis T., Solomou E.E. (2025). Diagnostic and Therapeutic Implications of the SUMOylation Pathway in Acute Myeloid Leukemia. Cancers.

[27] Senguttuvan N.B., Subramanian V., TR M., Sankaranarayanan K., Venkatesan V., Sadagopan T. (2025). Clonal Hematopoiesis of Indeterminate Potential and Cardiovascular Diseases: A Review. Indian Heart J..

[28] Reed S.C., Croessmann S., Park B.H. (2023). CHIP Happens: Clonal Hematopoiesis of Indeterminate Potential and Its Relationship to Solid Tumors. Clin. Cancer Res..

[29] Kinzhebay A., Salybekov A.A. (2025). The Role of Somatic Mutations in Ischemic Stroke: CHIP’s Impact on Vascular Health. Neurol. Int..

[30] Papa V., Marracino L., Fortini F., Rizzo P., Campo G., Vaccarezza M., Vieceli Dalla Sega F. (2020). Translating Evidence from Clonal Hematopoiesis to Cardiovascular Disease: A Systematic Review. J. Clin. Med..

[31] Libby P., Sidlow R., Lin A.E., Gupta D., Jones L.W., Moslehi J., Zeiher A., Jaiswal S., Schulz C., Blankstein R. (2019). Clonal Hematopoiesis: Crossroads of Aging, Cardiovascular Disease, and Cancer: JACC Review Topic of the Week. J. Am. Coll. Cardiol..

[32] Triantafyllou C., Peitz M., Fleischmann B.K., Rieck S. (2025). Generation of a homozygous DNMT3A knock-out hiPSC line for modeling of cardiovascular diseases associated with clonal hematopoiesis of indeterminate potential. Stem Cell Res..

[33] Watt S.M., Roubelakis M.G. (2025). Deciphering the Complexities of Adult Human Steady State and Stress-Induced Hematopoiesis: Progress and Challenges. Int. J. Mol. Sci..

[34] Jaiswal S., Fontanillas P., Flannick J., Manning A., Grauman P.V., Mar B.G., Lindsley R.C., Mermel C.H., Burtt N., Chavez A. (2014). Age-Related Clonal Hematopoiesis Associated with Adverse Outcomes. N. Engl. J. Med..

[35] Steensma D.P., Bejar R., Jaiswal S., Lindsley R.C., Sekeres M.A., Hasserjian R.P., Ebert B.L. (2015). Clonal Hematopoiesis of Indeterminate Potential and Its Distinction from Myelodysplastic Syndromes. Blood.

[36] Jaiswal S., Natarajan P., Silver A.J., Gibson C.J., Bick A.G., Shvartz E., McConkey M., Gupta N., Gabriel S., Ardissino D. (2017). Clonal Hematopoiesis and Risk of Atherosclerotic Cardiovascular Disease. N. Engl. J. Med..

[37] Yu B., Roberts M.B., Raffield L.M., Zekavat S.M., Nguyen N.Q.H., Biggs M.L., Brown M.R., Griffin G., Desai P., Correa A. (2021). Association of Clonal Hematopoiesis with Incident Heart Failure. J. Am. Coll. Cardiol..

[38] Dorsheimer L., Assmus B., Rasper T., Ortmann C.A., Ecke A., Abou-El-Ardat K., Schmid T., Brüne B., Wagner S., Serve H. (2019). Association of Mutations Contributing to Clonal Hematopoiesis with Prognosis in Chronic Ischemic Heart Failure. JAMA Cardiol..

[39] Sega F.V.D., Palumbo D., Fortini F., D’aGostino Y., Cimaglia P., Marracino L., Severi P., Strianese O., Tarallo R., Nassa G. (2022). Transcriptomic profiling of calcified aortic valves in clonal hematopoiesis of indeterminate potential carriers. Sci. Rep..

[40] Yang Y., Xia M., Jin Q., Bendahhou S., Shi J., Chen Y., Liang B., Lin J., Liu Y., Liu B. (2004). Identification of a KCNE2 Gain-of-Function Mutation in Patients with Familial Atrial Fibrillation. Am. J. Hum. Genet..

[41] Gollob M.H., Jones D.L., Krahn A.D., Danis L., Gong X.-Q., Shao Q., Liu X., Veinot J.P., Tang A.S.L., Stewart A.F.R. (2006). Somatic Mutations in the Connexin 40 Gene (GJA5) in Atrial Fibrillation. N. Engl. J. Med..

[42] Gutierrez A., Chung M.K. (2016). Genomics of Atrial Fibrillation. Curr. Cardiol. Rep..

[43] Karakasis P., Theofilis P., Lefkou E., Antoniadis A.P., Patoulias D., Korantzopoulos P., Fragakis N. (2025). Clonal Hematopoiesis of Indeterminate Potential and Atrial Fibrillation: Insights into Pathophysiology and Clinical Implications. Int. J. Mol. Sci..

[44] Liu J., Zhang N., Teng G., Tse G., Bai J., Lip G.Y.H., Liu T. (2025). Clonal Hematopoiesis of Indeterminate Potential and Atrial Fibrillation. Heart Rhythm..

[45] de la Nava A.M., González Mansilla A., González-Torrecilla E., Ávila P., Datino T., Bermejo J., Arenal Á., Fernández-Avilés F., Atienza F. (2021). Personalized Evaluation of Atrial Complexity of Patients Undergoing Atrial Fibrillation Ablation: A Clinical Computational Study. Biology.

[46] Chatzikalil E., Stergiou I.E., Papadakos S.P., Konstantinidis I., Theocharis S. (2024). The Clinical Relevance of the EPH/Ephrin Signaling Pathway in Pediatric Solid and Hematologic Malignancies. Int. J. Mol. Sci..

[47] Galeș L.N., Păun M.-A., Butnariu I., Simion L., Manolescu L.S.C., Trifănescu O.G., Anghel R.M. (2025). Next-Generation Sequencing in Oncology—A Guiding Compass for Targeted Therapy and Emerging Applications. Int. J. Mol. Sci..

[48] Kim K.I. (2023). Risk Stratification of Cardiovascular Disease according to Age Groups in New Prevention Guidelines: A Review. J. Lipid Atheroscler..

[49] Heimlich J.B., Bick A.G. (2022). Somatic Mutations in Cardiovascular Disease. Circ. Res..

[50] Zuo C., Fu D., Huang Y., Li J., Yang S., Cheng X., Zhang G., Ma T., Peng Q., Tan Y. (2025). Association of clonal hematopoiesis of indeterminate potential with cardiometabolic multimorbidity progression and mortality: A prospective study of UK Biobank. Eur. J. Med. Res..

[51] Winter S., Götze K.S., Hecker J.S., Metzeler K.H., Guezguez B., Woods K., Medyouf H., Schäffer A., Schmitz M., Wehner R. (2024). Clonal hematopoiesis and its impact on the aging osteo-hematopoietic niche. Leukemia.

[52] Kallai A., Ungvari A., Csaban D., Orfi Z., Lehoczki A., Harasztdombi J., Yabluchanskiy A., Benyó Z., Szappanos Á., Tarantini S. (2025). Clonal hematopoiesis of indeterminate potential (CHIP) in cerebromicrovascular aging: Implications for vascular contributions to cognitive impairment and dementia (VCID). GeroScience.

[53] Belizaire R., Wong W.J., Robinette M.L., Ebert B.L. (2023). Clonal Haematopoiesis and Dysregulation of the Immune System. Nat. Rev. Immunol..

[54] Li C., Zhang C., Li X. (2025). Clonal Hematopoiesis of Indeterminate Potential: Contribution to Disease and Promising Interventions. Mol. Cell. Biochem..

[55] Castellon X., Bogdanova V. (2016). Chronic Inflammatory Diseases and Endothelial Dysfunction. Aging Dis..

[56] Caiado F., Pietras E.M., Manz M.G. (2021). Inflammation as a Regulator of Hematopoietic Stem Cell Function in Disease, Aging, and Clonal Selection. J. Exp. Med..

[57] Bogeska R., Mikecin A.-M., Kaschutnig P., Fawaz M., Büchler-Schäff M., Le D., Ganuza M., Vollmer A., Paffenholz S.V., Asada N. (2022). Inflammatory Exposure Drives Long-Lived Impairment of Hematopoietic Stem Cell Self-Renewal Activity and Accelerated Aging. Cell Stem Cell.

[58] Ahn H.-J., An H.Y., Ryu G., Lim J., Sun C., Song H., Choi S.-Y., Lee H., Maurer T., Nachun D. (2024). Clonal Haematopoiesis of Indeterminate Potential and Atrial Fibrillation: An East Asian Cohort Study. Eur. Heart J..

[59] Mastrogeorgiou M., Chatzikalil E., Theocharis S., Papoudou-Bai A., Péoc’h M., Mobarki M., Karpathiou G. (2024). The Immune Microenvironment of Cancer of the Uterine Cervix. Histol. Histopathol..

[60] Zhao H., Wu L., Yan G., Chen Y., Zhou M., Wu Y., Li Y. (2021). Inflammation and Tumor Progression: Signaling Pathways and Targeted Intervention. Signal Transduct. Target. Ther..

[61] Hodel F., Naret O., Bonnet C., Brenner N., Bender N., Waterboer T., Marques-Vidal P., Vollenweider P., Fellay J. (2022). The Combined Impact of Persistent Infections and Human Genetic Variation on C-Reactive Protein Levels. BMC Med..

[62] Ninni S., Dombrowicz D., Kuznetsova T., Vicario R., Gao V., Molendi-Coste O., Haas J., Woitrain E., Coisne A., Neele A.E. (2023). Hematopoietic Somatic Mosaicism Is Associated with an Increased Risk of Postoperative Atrial Fibrillation. J. Am. Coll. Cardiol..

[63] Monnerat G., Lopez Alarcon M., Vasconcellos L., Hochman-Mendez C., Brasil G., Bassani R., Casis O., Malan D., Travassos L., Sepúlveda M. (2016). Macrophage-Dependent IL-1b Production Induces Cardiac Arrhythmias in Diabetic Mice. Nat. Commun..

[64] Chen Y.C., Voskoboinik A., La Gerche A., Marwick T.H., McMullen J.R. (2021). Prevention of Pathological Atrial Remodeling and Atrial Fibrillation. J. Am. Coll. Cardiol..

[65] Tall A., Fuster J. (2022). Clonal Hematopoiesis in Cardiovascular Disease and Therapeutic Implications. Nat. Cardiovasc. Res..

[66] Wang Y., Liu X., Shi H., Yu Y., Yu Y., Li M., Chen R. (2020). NLRP3 Inflammasome, an Immune-Inflammatory Target in Pathogenesis and Treatment of Cardiovascular Diseases. Clin. Transl. Med..

[67] Guarnera L., Jha B.K. (2024). TET2 Mutation as Prototypic Clonal Hematopoiesis Lesion. Semin. Hematol..

[68] Sano S., Oshima K., Wang Y., MacLauchlan S., Katanasaka Y., Sano M., Zuriaga M.A., Yoshiyama M., Goukassian D., Cooper M.A. (2018). Tet2-Mediated Clonal Hematopoiesis Accelerates Heart Failure Through a Mechanism Involving the IL-1β/NLRP3 Inflammasome. J. Am. Coll. Cardiol..

[69] Li J., Wang C., Liu J., Yu Y., Liu Y., Peng Q., Liu H., Guan X. (2021). A Feedback Loop: Interactions between Inflammatory Signals and Clonal Hematopoiesis in Cardiovascular Disease. Mol. Biol. Rep..

[70] Zhang S., Meng Y., Zhou L., Qiu L., Wang H., Su D., Zhang B., Chan K.-M., Han J. (2022). Targeting Epigenetic Regulators for Inflammation: Mechanisms and Intervention Therapy. MedComm.

[71] Fidler T.P., Xue C., Yalcinkaya M., Hardaway B., Abramowicz S., Xiao T., Liu W., Thomas D.G., Hajebrahimi M.A., Pircher J. (2021). The AIM2 Inflammasome Exacerbates Atherosclerosis in Clonal Haematopoiesis. Nature.

[72] Bejarano-Arosemena R., Martínez-Sellés M. (2023). Interatrial Block, Bayés Syndrome, Left Atrial Enlargement, and Atrial Failure. J. Clin. Med..

[73] Sagris M., Vardas E.P., Theofilis P., Antonopoulos A.S., Oikonomou E., Tousoulis D. (2022). Atrial Fibrillation: Pathogenesis, Predisposing Factors, and Genetics. Int. J. Mol. Sci..

[74] Pellman J., Lyon R.C., Sheikh F. (2010). Extracellular Matrix Remodeling in Atrial Fibrosis: Mechanisms and Implications in Atrial Fibrillation. J. Mol. Cell. Cardiol..

[75] Hinderer S., Schenke-Layland K. (2019). Cardiac Fibrosis—A Short Review of Causes and Therapeutic Strategies. Adv. Drug Deliv. Rev..

[76] Pradhan K., Neupane B., Niehues P., Kirschner M., Beier F., Kuo C.C., Hilbold E.A., Bär C., Thoma O.M., Waldner M. (2025). Telomere Length Is Associated with Adverse Atrial Remodeling in Patients with Atrial Fibrillation. J. Am. Heart Assoc..

[77] Dzeshka M.S., Lip G.Y.H., Snezhitskiy V., Shantsila E. (2015). Cardiac Fibrosis in Patients with Atrial Fibrillation: Mechanisms and Clinical Implications. J. Am. Coll. Cardiol..

[78] Chen R., Zhang H., Tang B., Luo Y., Yang Y., Zhong X., Chen S., Xu X., Huang S., Liu C. (2024). Macrophages in Cardiovascular Diseases: Molecular Mechanisms and Therapeutic Targets. Signal Transduct. Target. Ther..

[79] Ninni S., Dombrowicz D., de Winther M., Staels B., Montaigne D., Nattel S. (2024). Genetic Factors Altering Immune Responses in Atrial Fibrillation: JACC Review Topic of the Week. J. Am. Coll. Cardiol..

[80] Han H., Ge X., Komakula S.S.B., Desert R., Das S., Song Z., Chen W., Athavale D., Gaskell H., Lantvit D. (2023). Macrophage-Derived Osteopontin (SPP1) Protects from Nonalcoholic Steatohepatitis. Gastroenterology.

[81] Huang Y.-C., Wang C.-Y. (2021). Telomere Attrition and Clonal Hematopoiesis of Indeterminate Potential in Cardiovascular Disease. Int. J. Mol. Sci..

[82] Yao C., Veleva T., Scott L., Cao S., Li L., Chen G., Jeyabal P., Pan X., Alsina K., Abu-Taha I. (2018). Enhanced Cardiomyocyte NLRP3 Inflammasome Signaling Promotes Atrial Fibrillation. Circulation.

[83] Lin A.E., Bapat A.C., Xiao L., Niroula A., Ye J., Wong W.J., Agrawal M., Farady C.J., Boettcher A., Hergott C.B. (2024). Clonal Hematopoiesis of Indeterminate Potential with Loss of Tet2 Enhances Risk for Atrial Fibrillation through Nlrp3 Inflammasome Activation. Circulation.

[84] Nattel S., Harada M. (2014). Atrial Remodeling and Atrial Fibrillation: Recent Advances and Translational Perspectives. J. Am. Coll. Cardiol..

[85] Ihara K., Sugiyama K., Takahashi K., Yamazoe M., Sasano T., Furukawa T. (2018). Electrophysiological Assessment of Murine Atria with High-Resolution Optical Mapping. J. Vis. Exp. Jove.

[86] Landstrom A.P., Dobrev D., Wehrens X.H.T. (2017). Calcium Signaling and Cardiac Arrhythmias. Circ. Res..

[87] Hove-Madsen L., Llach A., Bayes-Genís A., Roura S., Font E.R., Arís A., Cinca J. (2004). Atrial Fibrillation Is Associated with Increased Spontaneous Calcium Release from the Sarcoplasmic Reticulum in Human Atrial Myocytes. Circulation.

[88] Lutsey P.L., Norby F.L., Alonso A., Cushman M., Chen L.Y., Michos E.D., Folsom A.R. (2018). Atrial Fibrillation and Venous Thromboembolism: Evidence of Bidirectionality in the Atherosclerosis Risk in Communities Study. J. Thromb. Haemost..

[89] Hobbs C.M., Manning H., Bennett C., Vasquez L., Severin S., Brain L., Mazharian A., Guerrero J.A., Li J., Soranzo N. (2013). JAK2V617F Leads to Intrinsic Changes in Platelet Formation and Reactivity in a Knock-in Mouse Model of Essential Thrombocythemia. Blood J. Am. Soc. Hematol..

[90] Arellano-Rodrigo E., Alvarez-Larrán A., Reverter J.C., Villamor N., Colomer D., Cervantes F. (2006). Increased Platelet and Leukocyte Activation as Contributing Mechanisms for Thrombosis in Essential Thrombocythemia and Correlation with the JAK2 Mutational Status. Haematologica.

[91] Liu W., Pircher J., Schuermans A., Ul Ain Q., Zhang Z., Honigberg M.C., Yalcinkaya M., Nakao T., Pournamadri A., Xiao T. (2024). Jak2 V617F Clonal Hematopoiesis Promotes Arterial Thrombosis via Platelet Activation and Cross Talk. Blood.

[92] Sozer S., Fiel M.I., Schiano T., Xu M., Mascarenhas J., Hoffman R. (2009). The Presence of JAK2V617F Mutation in the Liver Endothelial Cells of Patients with Budd-Chiari Syndrome. Blood J. Am. Soc. Hematol..

[93] Esmon C.T. (2005). The Interactions between Inflammation and Coagulation. Br. J. Haematol..

[94] Starikova E.A., Mammedova J.T., Rubinstein A.A., Sokolov A.V., Kudryavtsev I.V. (2025). Activation of the Coagulation Cascade as a Universal Danger Sign. Curr. Issues Mol. Biol..

[95] García-Escobar A., Lázaro-García R., Goicolea-Ruigómez J., González-Casal D., Fontenla-Cerezuela A., Soto N., González-Panizo J., Datino T., Pizarro G., Moreno R. (2024). Red Blood Cell Distribution Width Is a Biomarker of Red Cell Dysfunction Associated with High Systemic Inflammation and a Prognostic Marker in Heart Failure and Cardiovascular Disease: A Potential Predictor of Atrial Fibrillation Recurrence. High. Blood Press. Cardiovasc. Prev..

[96] Altieri C., Pisano C., Labriola V., Ferrante M., Porreca A., Nardi P., Bassano C., Buioni D., Greco E., Ruvolo G. (2022). Circulating Levels of Ferritin, RDW, PTLs as Predictive Biomarkers of Postoperative Atrial Fibrillation Risk after Cardiac Surgery in Extracorporeal Circulation. Int. J. Mol. Sci..

[97] Cha M.-J., Lee H.S., Kim H.M., Jung J.-H., Choi E.-K., Oh S. (2017). Association between Red Cell Distribution Width and Thromboembolic Events in Patients with Atrial Fibrillation. Eur. J. Intern. Med..

[98] Weymann A., Ali-Hasan-Al-Saegh S., Sabashnikov A., Popov A.F., Mirhosseini S.J., Liu T., Lotfaliani M., Sá M.P.B.O., Baker W.L.L., Yavuz S. (2017). Prediction of New-Onset and Recurrent Atrial Fibrillation by Complete Blood Count Tests: A Comprehensive Systematic Review with Meta-Analysis. Med. Sci. Monit. Basic Res..

[99] Vucinic V., Ruhnke L., Sockel K., Röhnert M.A., Backhaus D., Brauer D., Franke G.N., Niederwieser D., Bornhäuser M., Röllig C. (2021). The diagnostic red blood cell distribution width as a prognostic factor in acute myeloid leukemia. Blood Adv..

[100] Kar S.P., Quiros P.M., Gu M., Jiang T., Mitchell J., Langdon R., Iyer V., Barcena C., Vijayabaskar M.S., Fabre M.A. (2022). Genome-Wide Analyses of 200,453 Individuals Yield New Insights into the Causes and Consequences of Clonal Hematopoiesis. Nat. Genet..

[101] Schuermans A., Vlasschaert C., Nauffal V., Cho S.M.J., Uddin M.M., Nakao T., Niroula A., Klarqvist M.D.R., Weeks L.D., Lin A.E. (2024). Clonal Haematopoiesis of Indeterminate Potential Predicts Incident Cardiac Arrhythmias. Eur. Heart J..

[102] Hecker J.S., Hartmann L., Rivière J., Buck M.C., van der Garde M., Rothenberg-Thurley M., Fischer L., Winter S., Ksienzyk B., Ziemann F. (2021). CHIP and Hips: Clonal Hematopoiesis Is Common in Patients Undergoing Hip Arthroplasty and Is Associated with Autoimmune Disease. Blood.

[103] Evans M.A., Walsh K. (2022). Clonal Hematopoiesis, Somatic Mosaicism, and Age-Associated Disease. Physiol. Rev..

[104] Lueck C., Panagiota V., Dammann E., Gabdoulline R., Berliner D., Veltmann C., Heuser M., Beutel G., Ganser A., Eder M. (2022). Increased Late Noncardiac Nonrelapse Mortality in Patients with Atrial Fibrillation Diagnosed During Their Hospital Stay for Allogeneic Stem Cell Transplantation. Transplant. Cell. Ther..

[105] Zhao D., Wang Y., Wong N.D., Wang J. (2024). Impact of Aging on Cardiovascular Diseases. JACC Asia.

[106] Rodgers J.L., Jones J., Bolleddu S.I., Vanthenapalli S., Rodgers L.E., Shah K., Karia K., Panguluri S.K. (2019). Cardiovascular Risks Associated with Gender and Aging. J. Cardiovasc. Dev. Dis..

[107] Linz D., Gawalko M., Betz K., Hendriks J.M., Lip G.Y.H., Vinter N., Guo Y., Johnsen S. (2024). Atrial Fibrillation: Epidemiology, Screening and Digital Health. Lancet Reg. Health-Eur..

[108] Watson C.J., Papula A.L., Poon G.Y.P., Wong W.H., Young A.L., Druley T.E., Fisher D.S., Blundell J.R. (2020). The Evolutionary Dynamics and Fitness Landscape of Clonal Hematopoiesis. Science.

[109] Ferrone C.K., Blydt-Hansen M., Rauh M.J. (2020). Age-Associated TET2 Mutations: Common Drivers of Myeloid Dysfunction, Cancer and Cardiovascular Disease. Int. J. Mol. Sci..

[110] Kar R., Batra N., Riquelme M.A., Jiang J.X. (2012). Biological Role of Connexin Intercellular Channels and Hemichannels. Arch. Biochem. Biophys..

[111] Nachun D., Lu A., Bick A., Natarajan P., Weinstock J., Szeto M., Kathiresan S., Abecasis G., Taylor K., Guo X. (2021). Clonal Hematopoiesis Associated with Epigenetic Aging and Clinical Outcomes. Aging Cell.

[112] Arvanitakis K., Chatzikalil E., Kalopitas G., Patoulias D., Popovic D.S., Metallidis S., Kotsa K., Germanidis G., Koufakis T. (2024). Metabolic Dysfunction-Associated Steatotic Liver Disease and Polycystic Ovary Syndrome: A Complex Interplay. J. Clin. Med..

[113] Elliott A.D., Middeldorp M.E., Van Gelder I.C., Albert C.M., Sanders P. (2023). Epidemiology and Modifiable Risk Factors for Atrial Fibrillation. Nat. Rev. Cardiol..

[114] Levin M.G., Nakao T., Zekavat S.M., Koyama S., Bick A.G., Niroula A., Ebert B., Damrauer S.M., Natarajan P. (2022). Genetics of Smoking and Risk of Clonal Hematopoiesis. Sci. Rep..

[115] Bhattacharya R., Zekavat S., Uddin M., Pirruccello J., Niroula A., Gibson C., Griffin G., Libby P., Ebert B., Bick A. (2021). Association of Diet Quality With Prevalence of Clonal Hematopoiesis and Adverse Cardiovascular Events. JAMA Cardiol..

[116] Coccina F., Pierdomenico A., Ianni U., Rosa M., Luca A., Pirro D., Pizzicannella J., Trubiani O., Cipollone F., Renda G. (2020). Ambulatory Blood Pressure and Risk of New-onset Atrial Fibrillation in Treated Hypertensive Patients. J. Clin. Hypertens..

[117] Tobias D.K., Manning A.K., Wessel J., Raghavan S., Westerman K.E., Bick A.G., Dicorpo D., Whitsel E.A., Collins J., Correa A. (2023). Clonal Hematopoiesis of Indeterminate Potential (CHIP) and Incident Type 2 Diabetes Risk. Diabetes Care.

[118] Vilariño-García T., Polonio-González M.L., Pérez-Pérez A., Ribalta J., Arrieta F., Aguilar M., Obaya J.C., Gimeno-Orna J.A., Iglesias P., Navarro J. (2024). Role of Leptin in Obesity, Cardiovascular Disease, and Type 2 Diabetes. Int. J. Mol. Sci..

[119] Challen G., Sun D., Jeong M., Luo M., Jelinek J., Vasanthakumar A., Meissner A., Issa J.-P., Godley L., Li W. (2011). Dnmt3a Is Essential for Hematopoietic Stem Cell Differentiation. Blood.

[120] Okano M., Bell D.W., Haber D.A., Li E. (1999). DNA Methyltransferases Dnmt3a and Dnmt3b Are Essential for De Novo Methylation and Mammalian Development. Cell.

[121] Russler-Germain D.A., Spencer D.H., Young M.A., Lamprecht T.L., Miller C.A., Fulton R., Meyer M.R., Erdmann-Gilmore P., Townsend R.R., Wilson R.K. (2014). The R882H DNMT3A Mutation Associated with AML Dominantly Inhibits Wild-Type DNMT3A by Blocking Its Ability to Form Active Tetramers. Cancer Cell.

[122] Hormaechea-Agulla D., Matatall K., Le D., Kain B., Long X., Kuś P., Jaksik R., Challen G., Kimmel M., King K. (2021). Chronic Infection Drives Dnmt3a-Loss-of-Function Clonal Hematopoiesis via IFNγ Signaling. Cell Stem Cell.

[123] Gleitz H.F.E., Schneider R.K. (2023). “ASXL1”-Erating Inflammation and Bone Marrow Fibrosis in Myeloproliferative Neoplasms. Haematologica.

[124] Shao J., Liu J., Zuo S. (2022). Roles of Epigenetics in Cardiac Fibroblast Activation and Fibrosis. Cells.

[125] Rauch P., Gopakumar J., Silver A., Nachun D., Ahmad H., McConkey M., Nakao T., Bossé M., Rentz T., Gonzalez N. (2023). Loss-of-Function Mutations in Dnmt3a and Tet2 Lead to Accelerated Atherosclerosis and Concordant Macrophage Phenotypes. Nat. Cardiovasc. Res..

[126] Yagi M., Kabata M., Tanaka A., Ukai T., Ohta S., Nakabayashi K., Shimizu M., Hata K., Meissner A., Yamamoto T. (2020). Identification of Distinct Loci for de Novo DNA Methylation by DNMT3A and DNMT3B during Mammalian Development. Nat. Commun..

[127] Zeng H., Liu X., Li Y., Zhou C. (2021). Abnormal Expressions of Plasma DNMT3A in Elderly Patients with Recurrent Atrial Fibrillation. Int. J. Clin. Exp. Med..

[128] Tulstrup M., Soerensen M., Hansen J.W., Gillberg L., Needhamsen M., Kaastrup K., Helin K., Christensen K., Weischenfeldt J., Grønbæk K. (2021). TET2 Mutations Are Associated with Hypermethylation at Key Regulatory Enhancers in Normal and Malignant Hematopoiesis. Nat. Commun..

[129] Rasmussen K., Berest I., Kessler S., Nishimura K., Simón-Carrasco L., Vassiliou G., Pedersen M., Christensen J., Zaugg J., Helin K. (2019). TET2 Binding to Enhancers Facilitates Transcription Factor Recruitment in Hematopoietic Cells. Genome Res..

[130] Gao Q., Shen K., Xiao M. (2024). TET2 Mutation in Acute Myeloid Leukemia: Biology, Clinical Significance, and Therapeutic Insights. Clin. Epigenet..

[131] Pan X., Chang Y.-J., Ruan G., Zhou S., Jiang H., Jiang Q., Huang X., Zhao X. (2024). TET2 Mutations Contribute to Adverse Prognosis in Acute Myeloid Leukemia (AML): Results from a Comprehensive Analysis of 502 AML Cases and the Beat AML Public Database. Clin. Exp. Med..

[132] Woo J., Lu D., Lewandowski A., Xu H., Serrano P., Healey M., Yates D.P., Beste M.T., Libby P., Ridker P.M. (2023). Effects of IL-1β Inhibition on Anemia and Clonal Hematopoiesis in the Randomized CANTOS Trial. Blood Adv..

[133] Asada S., Fujino T., Goyama S., Kitamura T. (2019). The Role of ASXL1 in Hematopoiesis and Myeloid Malignancies. Cell. Mol. Life Sci..

[134] Haring B., Wissel S., Manson J.E. (2022). Somatic Mutations and Clonal Hematopoiesis as Drivers of Age-Related Cardiovascular Risk. Curr. Cardiol. Rep..

[135] Florez M.A., Tran B.T., Wathan T.K., DeGregori J., Pietras E.M., King K.Y. (2022). Clonal Hematopoiesis: Mutation-Specific Adaptation to Environmental Change. Cell Stem Cell.

[136] Min K., Polizio A., Kour A., Thel M., Walsh K. (2022). Experimental ASXL1-Mediated Clonal Hematopoiesis Promotes Inflammation and Accelerates Heart Failure. J. Am. Heart Assoc..

[137] Bader M.S., Meyer S.C. (2022). JAK2 in Myeloproliferative Neoplasms: Still a Protagonist. Pharmaceuticals.

[138] Perner F., Perner C., Ernst T., Heidel F.H. (2019). Roles of JAK2 in Aging, Inflammation, Hematopoiesis and Malignant Transformation. Cells.

[139] Cordua S., Kjaer L., Skov V., Pallisgaard N., Hasselbalch H., Ellervik C. (2019). Prevalence and Phenotypes of JAK2 V617F and Calreticulin Mutations in a Danish General Population. Blood.

[140] Bick A.G., Weinstock J.S., Nandakumar S.K., Fulco C.P., Bao E.L., Zekavat S.M., Szeto M.D., Liao X., Leventhal M.J., Nasser J. (2020). Inherited Causes of Clonal Haematopoiesis in 97,691 Whole Genomes. Nature.

[141] Sano S., Wang Y., Ogawa H., Horitani K., Sano M., Polizio A.H., Kour A., Yura Y., Doviak H., Walsh K. (2021). TP53-Mediated Therapy-Related Clonal Hematopoiesis Contributes to Doxorubicin-Induced Cardiomyopathy by Augmenting a Neutrophil-Mediated Cytotoxic Response. JCI Insight.

[142] Sun Y., Yu Y., Cai L., Yu B., Xiao W., Tan X., Wang Y., Lu Y., Wang N. (2025). Clonal Hematopoiesis of Indeterminate Potential, Health Indicators, and Risk of Cardiovascular Diseases among Patients with Diabetes: A Prospective Cohort Study. Cardiovasc. Diabetol..

[143] Nishida A., Andoh A. (2025). The Role of Inflammation in Cancer: Mechanisms of Tumor Initiation, Progression, and Metastasis. Cells.

[144] Delaporta P., Chatzikalil E., Kyriakopoulou D., Berdalli S., Chouliara V., Hatzieleftheriou M.-I., Mylona S., Kattamis A. (2024). Abrupt Increases in Ferritin Levels May Indicate a Malignant Process and Not Changes in Iron Overload in Thalassemic Patients. Blood.

[145] Chatzikalil E., Arvanitakis K., Kalopitas G., Florentin M., Germanidis G., Koufakis T., Solomou E.E. (2025). Hepatic Iron Overload and Hepatocellular Carcinoma: New Insights into Pathophysiological Mechanisms and Therapeutic Approaches. Cancers.

[146] Cacciatore S., Andaloro S., Bernardi M., Oterino Manzanas A., Spadafora L., Figliozzi S., Asher E., Rana J.S., Ecarnot F., Gragnano F. (2025). Chronic Inflammatory Diseases and Cardiovascular Risk: Current Insights and Future Strategies for Optimal Management. Int. J. Mol. Sci..

[147] Tan H., Jiang H., Wang S. (2025). Biomarkers in Clonal Haematopoiesis of Indeterminate Potential (CHIP) Linking Cardiovascular Diseases, Myeloid Neoplasms and Inflammation. Ann. Hematol..

[148] Bulnes J.F., González L., Velásquez L., Orellana M.P., Venturelli P.M., Martínez G. (2024). Role of Inflammation and Evidence for the Use of Colchicine in Patients with Acute Coronary Syndrome. Front. Cardiovasc. Med..

[149] Nidorf S.M., Fiolet A.T.L., Mosterd A., Eikelboom J.W., Schut A., Opstal T.S.J., The S.H.K., Xu X.-F., Ireland M.A., Lenderink T. (2020). Colchicine in Patients with Chronic Coronary Disease. N. Engl. J. Med..

[150] Zuriaga M.A., Yu Z., Matesanz N., Truong B., Ramos-Neble B.L., Asensio-López M.C., Uddin M.M., Nakao T., Niroula A., Zorita V. (2024). Colchicine Prevents Accelerated Atherosclerosis in TET2-Mutant Clonal Haematopoiesis. Eur. Heart J..

[151] Bick A.G., Pirruccello J.P., Griffin G.K., Gupta N., Gabriel S., Saleheen D., Libby P., Kathiresan S., Natarajan P. (2020). Genetic Interleukin 6 Signaling Deficiency Attenuates Cardiovascular Risk in Clonal Hematopoiesis. Circulation.

[152] Ridker P.M., Devalaraja M., Baeres F.M.M., Engelmann M.D.M., Hovingh G.K., Ivkovic M., Lo L., Kling D., Pergola P., Raj D. (2021). IL-6 Inhibition with Ziltivekimab in Patients at High Atherosclerotic Risk (RESCUE): A Double-Blind, Randomised, Placebo-Controlled, Phase 2 Trial. Lancet.

[153] Fedele D., Alvarez M.C., Maida A., Vasumini N., Amicone S., Canton L., Di Leo M., Basile M., Manaresi T., Angeli F. (2025). Prevention of atrial fibrillation with SGLT2 inhibitors across the spectrum of cardiovascular disorders: A meta-analysis of randomised controlled trials. Eur. Heart J. Cardiovasc. Pharmacother..

[154] Adamstein N.H., Cornel J.H., Davidson M., Libby P., De Remigis A., Jensen C., Ekström K., Ridker P.M. (2023). Association of Interleukin 6 Inhibition with Ziltivekimab and the Neutrophil-Lymphocyte Ratio: A Secondary Analysis of the RESCUE Clinical Trial. JAMA Cardiol..

[155] Fennell K.A., Bell C.C., Dawson M.A. (2019). Epigenetic Therapies in Acute Myeloid Leukemia: Where to from Here?. Blood.

[156] Arvanitakis K., Papadakos S.P., Vakadaris G., Chatzikalil E., Stergiou I.E., Kalopitas G., Theocharis S., Germanidis G. (2024). Shedding Light on the Role of LAG-3 in Hepatocellular Carcinoma: Unraveling Immunomodulatory Pathways. Hepatoma Res..

[157] Papadakos S.P., Chatzikalil E., Arvanitakis K., Vakadaris G., Stergiou I.E., Koutsompina M.-L., Argyrou A., Lekakis V., Konstantinidis I., Germanidis G. (2024). Understanding the Role of Connexins in Hepatocellular Carcinoma: Molecular and Prognostic Implications. Cancers.

[158] Nakato R., Sakata T. (2021). Methods for ChIP-Seq Analysis: A Practical Workflow and Advanced Applications. Methods.

[159] Zou Z., Iwata M., Yamanishi Y., Oki S. (2022). Epigenetic Landscape of Drug Responses Revealed through Large-Scale ChIP-Seq Data Analyses. BMC Bioinform..

